# Does the Credit Cycle Have an Impact on Happiness?

**DOI:** 10.3390/ijerph17010183

**Published:** 2019-12-26

**Authors:** Tinghui Li, Junhao Zhong, Mark Xu

**Affiliations:** 1 School of Economics and Statistics, Guangzhou University, Guangzhou 510006, China; lith@gzhu.edu.cn; 2 Portsmouth Business School, University of Portsmouth, Portsmouth PO1 3DE, UK; Mark.Xu@port.ac.uk

**Keywords:** the credit cycle, happiness, moderating effect, credit expansion, credit recession

## Abstract

The 2008 international financial crisis triggered a heated discussion of the relationship between public health and the economic environment. We test the relationship between the credit cycle and happiness using the fixed effects model and explore the transmission channels between them by adding the moderating effect. The results show the following empirical regularities. First, the credit cycle has a negative correlation with happiness. This means that credit growth will reduce the overall happiness score in a country/region. Second, the transmission channels between the credit cycle and happiness are different during credit expansion and recession. Life expectancy and generosity can moderate the relationship between the credit cycle and happiness only during credit expansion. GDP per capita can moderate this relationship only during credit recession. Social support, freedom, and positive affect can moderate this relationship throughout the credit cycle. Third, the total impact of the credit cycle on happiness will become positive by the changes in the moderating effects. In general, we can improve subjective well-being if one of the following five conditions holds: (1) with the adequate support from the family and society, (2) with enough freedom, (3) with social generosity, (4) with a positive and optimistic outlook, and (5) with a high level of GDP per capita.

## 1. Introduction

Psychologist and economists are now trying hard to understand the effect of the subprime mortgage crisis on happiness. The happiness index of most countries fell quite substantially during the 2007–2008 financial crash. Particularly, regions in Western Europe, North America, and South Asia suffered from substantial declines in the happiness index between pre- and post-crisis. They have fallen to a “happiness trough” in 2009 (for more detailed analysis of the available global data on national happiness see World Happiness Reports 2019: https://s3.amazonaws.com/happiness-report). On average, credit-to-GDP reached a peak in the third quarter of 2009. Noteworthily, the credit cycle can be explained by the credit-to-GDP based on most of the literature [1,2,3]. The credit data from 42 economies provided by Bank for International Settlements (BIS) indicate that the national average of credit-to-GDP reached a historical peak in the third quarter of 2009 (Source: BIS credit-to-GDP gap statistics). Both results appear to provide evidence on the credit-happiness link. On the other side, the study of happiness in what has become known as the “economics of happiness” has led to much debate over the link between national economic conditions (or income inequality) and subjective well-being [4,5,6,7,8,9]. Given that credit spreads reflect the state of the business cycle ahead [10,11], the fluctuation in the credit cycle would likely cause the increase and decrease in the happiness index as well. For this reason, examining the relationship between the credit cycle and happiness, our chief concern in this paper, is important.

Many studies have confirmed that the credit cycle booms and busts hold risks for the economy. Housing demand increases and house prices rise are well explained by credit expansion [12,13,14,15]. Other than this, excess credit growth aggravated the harmful effects of financial stress and risks on the real economy. Specifically, a 1% increase in pre-crisis lending translates into a 0.2% increase in the cumulative loss in real output [16]. One distinguishing reason for this effect is that while the increase in the supply of bank loans increases the level of non-performing loans, it does not lead to higher profitability [17,18,19,20]. In addition, flows of bank loans to the non-financial private sector drive the build-up of current account imbalances in the deficit countries [21,22,23,24]. Thus, excess credit growth may crowd out real economic growth [25,26,27]. The credit contraction depressed investment, employment, and sales growth of firms and it explains most of the negative real effects [28,29]. The investment of bank-dependent industries falls substantially [30]. During the Great Depression, the credit crunch explained a substantial fraction of the aggregate decline in employment [31].

In recent decades the research on happiness has been linked to economics [6]. The attention on the relationship between happiness and income is a part of a broad, ongoing controversy. Many of the basic theory of happiness-income in existing literature follow Easterlin [32,33] and assign income a central role in affecting happiness in the short term. They state that at a point in time happiness varies directly with income both among and within nations, but over time happiness does not trend upward as income continues to grow (the strength of the association diminishes with higher income). The above research has also been questioned. Stevenson and Wolfers [34] find that economic growth associated with rising happiness by examining the relationship between changes in happiness and income over time within countries. Some researchers believe that the degree of wealth of the country should be considered when examining the relationship between personal income and happiness [4,35,36,37,38]. Beyond income, however, many social scientists have documented that happiness is associated with the person’s state of health and ageing [39,40], income inequality [41,42], natural environments [43,44], level of education [45], financial crisis [46], and quality of governance [47,48].

Extant studies on the effect of credit on happiness have primarily focused on the empirical investigation of the micro-level personal and household debt. While personal (or household) debt and happiness are robustly and negatively associated in cross-sectional studies [49], a random-effects meta-analysis of seven studies showed a weak link between debt and happiness (r=−0.07) [50], resulting in considerable debate on whether personal debt substantially matters for mental health [51,52,53]. Many social scientists, however, have documented that the impact of different types of debt on subjective well-being is heterogeneous. One intriguing result is that happiness was negatively affected by subjective debt (worry about debt), but was statistically insignificant by objective debt (debt-to-income ratio) [54]. The other is that the different sources of housing debt have different effects on happiness, and only nonbank housing debt significantly reduces happiness [55]. This attention on the relationship between personal (or household) debt and happiness at the micro-level is also part of an ongoing controversy on whether expansion and recession of credit lead to a lower level of happiness. Moreover, given the prevalence of debt in a modern economy, these discussions of whether debt decrease can lead to happiness omit a critical macro-level variable—the credit cycle.

Our study makes three contributions to the existing literature on the credit cycle and happiness. First, we test and demonstrate the negative correlation between the credit cycle and happiness. This differs from the previous literature in that we analyze the impact of the credit on happiness from a macro perspective. For policy makers who are committed to improving national happiness scores, it is an interesting question to specify how to improve subjective well-being based on the negative correlation between the credit cycle and happiness. Second, we explore the transmission channels of the credit cycle on happiness. Several moderating variables have been found to moderate the relationship between the credit cycle and happiness in our study. However, the moderating effects between the credit cycle and happiness are different during credit expansion and recession. Three, we also found an interesting result, that is, the negative impact of the credit cycle on happiness will become positive as the value of the moderating variable changes. During credit growth or recession, this finding tells us that we can improve the happiness score by moderating variables. This can effectively reduce the negative impact of the credit fluctuations on subjective well-being.

The remainder of this paper is organized as follows. Section 2 develops our research hypotheses. Section 3 contains methodology and data source. Section 4 empirically examines the impact of the credit cycle on happiness. Section 5 further studies the moderating effect between the credit cycle and happiness. Finally, Section 6 concludes our paper and provides some policy recommendations.

## 2. Hypothesis

### 2.1. Credit Cycle and Happiness

The most prominent study to date focused on the measurement of happiness and argued that happiness relates to life evaluation, positive affect, and negative affect [56]. Many studies have examined the association between happiness and life satisfaction and established that life satisfaction is one component of happiness. However, happiness and life satisfaction are empirically and conceptually distinct. Life satisfaction might be characterized by more profound enjoyment and achievement in life than happiness [57]. Furthermore, positive and negative affect can directly elicit feelings, such as happiness and sadness.

Credit growth can indirectly affect the quality of life of residents by rising house prices and inflation, etc. The ultimate impact of credit growth is a decline in happiness. For example, throughout the 1990s and early 2000s, the Icelandic banking industry grew rapidly. Bank loan size in Iceland shows a long-term growth trend, leading to the result of rising house prices and increased consumption. As credit continues to expand, the happiness of Icelandic adults continues to decline [58]. During a credit contraction, bank loans will reduce happiness by increasing residents’ financial stress and worries. A study of college students’ credit card use found that satisfaction of one’s own financial situation can improve sense of material well-being [59,60]. In addition, objective debt (credit card debt) was positively associated with negative affect (depression, exhaustion, sadness) [61]. In this sense, we proposed that the credit cycle could impact on happiness. Therefore, it is reasonable to hypothesize that:

**Hypothesize 1** **(H1).** *A negative correlation exists between the credit cycle and happiness.*

### 2.2. Moderating Hypothesis

A healthy life and life expectancy are strongly linked to happiness, and this correlation could increase with age. The prevalence of chronic diseases increases with age. As life expectancy increases and treatments for life-threatening diseases become more effective, the issue of maintaining health at an advanced age becomes more and more important [39]. The Gallup World Poll conducted ongoing surveys in more than 160 countries and found that in high-income, English-speaking countries, evaluable well-being has a U-shaped relationship with age. What is shocking is that the minimum level of happiness in these countries is in 45–54 years old. But this pattern is not universal. A study challenges the notion that wellbeing is U-shaped throughout the life course and underscores the critical role of mobility across wellbeing domains in later life [62]. The above shows that there is a significant correlation between happiness and life expectancy. Happiness is affected by many factors other than health.

Research in the field of psychology suggests that generous behavior show strong increases in self-reported happiness [63,64]. There is a model of a positive feedback loop between prosocial spending and happiness. Prosocial spending leads to an increase in happiness. Happiness, in turn, increases the likelihood of engaging in future acts of prosocial spending [65]. Park et al. [66] presented a mechanistic understanding of the neural processes linking generosity and happiness. Generous decisions engage the temporo-parietal junction (TPJ) in those who spend money on others more than in those who spend on themselves and differentially modulate the connectivity between TPJ and ventral striatum. What counts is that the activity of ventral striatum is directly related to changes in happiness. From the perspective of psychology or sociology, generosity behavior can increase happiness.

Credit expansion may affect both life expectancy and generosity, as it may impinge on the alcohol-related disorders and national mood, such as anxiety, worry, and testiness. For example, a study showed that credit expansion was an important factor that had a negative impact on workers’ mental health. Furthermore, problems related to the subprime crisis may have also affected the general health of workers by increasing the risk of such health problems as cardiovascular and respiratory diseases [67]. In addition, credit expansion can also influence generous behavior and prosocial spending. Credit growth plays an important role in stimulating economic growth and is strongly associated with faster economic growth [68,69]. An interesting experiment by Kellner et al. [70] showed that when people knew their income would increase, their donations would be higher. This means that credit growth indirectly increases the generosity of society by stimulating economic growth. In this sense, we propose that the credit cycle could trigger happiness during the period of credit expansion via life expectancy and generosity. Therefore, we formulate the following hypothesis:

**Hypothesize H2a** **(H2a).** *During the period of credit expansion, life expectancy and generosity moderate the relationship between the credit cycle and happiness.*

There is an important viewpoint in mainstream welfare economics that suggests that higher levels of economic development are associated with higher levels of well-being. Further research finds that the richer the society, the less do gains in economic growth confer gains in individuals’ happiness [71,72]. For example, Wu and Li [73] provide empirical evidence that local economic growth has a positive effect on individuals’ life satisfaction. However, there is more and more evidence that across countries, the relationship between GDP per capita and subjective wellbeing is roughly log-linear [34,74,75].

Credit contraction can be an important predictor of economic recession. López-Salido et al. [11] show that elevated credit-market sentiment in year t−2 is associated with a decline in economic activity in years t and t+1 using U.S. data from 1929 to 2015. It is believed that credit contraction may influence economic development. Our measure of economic development is the natural logarithm of real GDP per capita, measured at purchasing power parity. As Jebb et al. [7] have noted, all analyses were done using the natural logarithm of GDP per capita, which is standard practice because the relationship between happiness and GDP per capita is known to be log-linear. In this sense, we propose that the credit cycle could trigger happiness during the period of credit contraction via GDP per capita. Therefore, we formulate the following hypothesis:

**Hypothesize H2b** **(H2b).** *During the period of credit contraction, GDP per capita moderates the relationship between the credit cycle and happiness.*

Social support, freedom and positive affect are three of the important predictors of happiness. People with satisfactory social relationships are more likely to be happy, less sorrowful, and more satisfied with life than those who do not have a satisfactory social relationship [76]. Alternatively, countries with a higher level of economic freedom experienced a consistently higher level of happiness [77]. Furthermore, positive emotions have been shown to benefit from optimistic perceptions, even if these perceptions are illusory [78].

A large behavioral literature shows that changes in social support, freedom, and positive affect are associated with the fluctuation in the credit cycle. First, the credit collapse during the global financial crisis of 2008 led to an increase in unemployment. Unemployment leads to increased social isolation that either directly affects mental health [79]. For example, Gudmundsdóttir et al. [58] demonstrate that one of the important reasons why the unemployed are prone to mental disorders during the crisis is the decline in support from society and the family. Second, economic freedom fall is affected by credit contraction and worsen the economic environment. The undesirable result is a decline in happiness. Karfakis [80] shows that the credit collapse during the global financial crisis of 2008 seems to be one of the forces which are responsible for the collapse of the real economy. The global economic downturn of 2008–2009 led to a decrease in income, and there is a positive association between income and economic freedom. Furthermore, some of the most consistent findings in existing literature are that democracy and political freedom are positively associated with economic freedom; and inequality is negatively related to economic freedom [81]. The study of Bjørnskov [82] suggests that economic freedom is robustly associated with smaller peak-to-trough ratios and shorter recovery time. This means that the fluctuation in the credit cycle can lead to a decrease in happiness by reducing economic freedom. Third, fluctuation of the credit cycle was positively associated with financial anxiety and it has reduced positive affect [83]. This indicates that fluctuation of the credit cycle can lead to a decline in happiness through the channel of positive affect. In this sense, we propose that the credit cycle could trigger happiness whether the credit cycle is during expansion or recession phases via social support, freedom and positive affect. Therefore, we formulate the following hypothesis:

**Hypothesize H2c** **(H2c).** *Social support, freedom, and positive affect moderate the relationship between the credit cycle and happiness whether the credit cycle is during expansion or recession phases.*

It is common knowledge that the extent of happiness might change as many important indicators change. A study using more than 76,000 bank-transaction records found that if individuals spend more on products that match their personality, people report higher levels of happiness [84]. Gonza and Burger [85] suggest that inter-regional and inter-cultural differences are additional determinants of happiness during the 2008 financial crisis. This would guide us to track the change of influential factors throughout the credit cycle and hence test their relationship between influential factors and happiness. In the context of fluctuations in the credit cycle, we can reasonably assume that the important factors affecting happiness—life expectancy, generosity, GDP per capita, social support, freedom, and positive affect—can be artificially regulated, and the negative impact of the credit cycle on happiness will turn into a positive impact. Therefore, the most heuristic prediction of this study is as follows:

**Hypothesize H3** **(H3).** *The credit cycle has a chance to increase happiness once the value of moderating variable changes.*

## 3. Methods and Data

### 3.1. Method

Panel data models are widely used in the study of the influencing factors of happiness. It offers in reflecting the variation of happiness both over time and across economies. Among the three kinds of panel data regression models—the fixed effects model, the random effects model, and the mixed effects model. We proceed with firstly an F-test to identify if either mixed or fixed effects are preferred for the regression specification. The null hypothesis of the F-test is rejected at the 1% level. Then, the Hausman test is commonly used to test the applicability of the fixed and random effects model. In our study, the null hypothesis of the Hausman test is rejected at the 1% level. Thus, we applied a fixed effects model to explore the effects of the credit cycle on happiness. In order to study the impact of the credit cycle on the reported happiness and particularly the varying effect of credit expansion and contraction on happiness, we estimate the following regression specification:

(1) $$Happiness_{ct}=\alpha Creditcycle_{ct}+\Gamma Control_{ct}+C_{c}+T_{t}+\mu_{ct},$$

where $Happiness_{ct}$ is the dependent variable, c=country and $t=\mathrm{time},\;\mathrm{and}\;Creditcycle_{ct}$ denotes the credit cycle. $Control_{ct}$ is a vector of control variable. We also include a set of economy-fixed effects, $C_{c}$, and year-fixed effects, $T_{t}$ $\mu_{ct}$ is idiosyncratic an error term.

To test for any asymmetric effects of the credit cycle, we then fit other panel regression specification (2) and (3) that introduces expansion or recession phase of the credit cycle, such that:

(2) $$Happiness_{ct}=\alpha Expansion_{ct}+\Gamma Control_{ct}+C_{c}+T_{t}+\mu_{ct},$$

(3) $$\;Happiness_{ct}=\alpha Recession_{ct}+\Gamma Control_{ct}+C_{c}+T_{t}+\mu_{ct},\;$$

where $Expansion_{ct}$ denotes expansion phase of the credit cycle and $Recession_{ct}$ denotes recession phase of the credit cycle. Following the setting of Gonza and Burger [85] for expansion and recession, the variables $Expansion_{ct}$ and $Recession_{ct}$ are replaced by a continuous variable measuring the growth rate of credit-to-GDP to distinguish between the credit cycle of a different amplitude. When the growth rate of credit-to-GDP is greater than or equal to 0, this period represents an expansion period, otherwise, it is a recession period. We can analyze the effects of credit cycles on happiness and heterogeneity effect through the coefficients α of regression specification (1)–(3).

As pointed out in the moderating hypothesis of our study, moderating variables will moderate the relationship between the credit cycle and happiness in different phases. Thus, we would expect to test a moderating effect between the credit cycle and happiness considering different moderating variable. In further research, we therefore investigate whether the relationship between the credit cycle and happiness changes over time because of the moderating hypothesis of our study. For this purpose, we estimate the following regression specification:

(4) $$Happiness_{ct}=\alpha Creditcycle_{ct}+\beta Moderate_{ct}+\lambda Creditcycle_{ct}\times Moderate_{ct}+\Gamma Control_{ct}+C_{c}+T_{t}+\mu_{ct},$$

where $Moderate_{ct}$ is a moderating variable and $Creditcycle_{ct}\times Moderate_{ct}$ is an interaction term. We can analyze the moderating effects between the credit cycles and happiness through the regression specification (4). However, we make a simple conversion of the regression specification (4) to more directly express the total effect of the credit cycle on happiness after we add a moderating variable:

(5) $$Happiness_{ct}=\left(\alpha +\lambda Moderate_{ct}\right)\times Creditcycle_{ct}+\beta Moderate_{ct}+\Gamma Control_{ct}+C_{c}+T_{t}+\mu_{ct},$$

where $\alpha +\lambda Moderate_{ct}$ is the total impact that denotes the direct and the moderating effects in the relationship between the credit cycles and happiness.

### 3.2. Variables and Data Source

We seek to investigate the basic question raised earlier regarding the influences of the credit cycle on happiness. For this purpose, the selected variables and the following database has been collected from these sources as shown in Table 1. Happiness is an explained variable. In this study, happiness is measured by the national-level average scores in a country/region for subjective well-being taken from the World Happiness Report. There is skepticism about interpreting happiness scores as cardinal numbers, but Kahneman et al. [86], Frey and Stutzer [48] showed that this may be less problematic at a practical level than at the theoretical level. Di Tella et al. [87], Ferrer-i-Carbonell and Frijters [40] also indicated that assuming cordiality or cardinality of happiness scores had little impact on empirical results. The credit cycle is an explanatory variable. Noteworthily, the credit cycle can be explained by the credit-to-GDP based on most of the literature [1,2,3]. We follow the mainstream literature on the setting of the credit cycle, which uses credit-to-GDP as the proxy variable of the credit cycle. They found that the credit-to-GDP best fits the credit cycle of a country/region. Given the control variables and the moderating variables, we refer to the six key variables mentioned in the ‘World Happiness Report 2019′ to explaining the full sample of national annual average happiness scores over the whole period 2006–2018. These six variables are GDP per capita, social support, healthy life expectancy, freedom, generosity, and the absence of corruption [88]. Moreover, we also added the positive and negative affect which are the main forces that influence happiness.

This study uses three questions for measuring variable “positive affect”: individual indicate “did you experience ‘happiness’ during a lot of the day yesterday?”, “did you smile or laugh a lot yesterday?” and “did you experience ‘enjoyment’ during a lot of the day yesterday?”, respectively [88]. If an interviewee answers “Yes”, then it = 1 point, otherwise, 0 points. The final data is the arithmetic mean of the scores for these three questions. Similarly, the measurement of the variable “negative emotion” can be explained following three questions: “did you experience ‘worry’ during a lot of the day yesterday?”, “did you experience ‘sadness’ during a lot of the day yesterday?” and “did you experience ‘anger’ during a lot of the day yesterday?”, respectively.

## 4. The Impact of the Credit Cycle on Happiness

### 4.1. Descriptive Statistics and Stationarity Test

Below, a system of summary statistics and stationarity tests for all variables will be built. This study examines the effects of the credit cycle on happiness of nationally representative samples in 42 mainly economies between 2006 and 2018. The remaining economies are excluded due to the insufficient number of observations. In particular, the economies included in the study are: Argentina, Australia, Austria, Belgium, Brazil, Canada, Chile, China, Colombia, Czech Republic, Denmark, Finland, France, Germany, Greece, Hong Kong S.A.R. of China, Hungary, India, Indonesia, Ireland, Israel, Italy, Japan, Luxembourg, Malaysia, Mexico, Netherlands, New Zealand, Norway, Poland, Portugal, Russia, Saudi Arabia, Singapore, South Africa, Spain, Sweden, Switzerland, Thailand, Turkey, United Kingdom, and the United States. Table 2 is descriptive statistics that provide a summary on a variety of variables, including happiness, the credit cycle, GDP per capita, healthy life expectancy, social support, freedom, generosity, corruption perception, positive affect, and negative affect.

Before studying the relationship between happiness and the credit cycle, the unit root test is performed to check whether the variables used in this article are stationary or integrated of the same order. The data in our study is unbalanced panel data because some observations are missing in some countries. Therefore, we choose the augmented Dickey-Fuller (ADF) unit-root tests test [89] and the Phillips–Perron unit-root tests [90]. The results in levels are reported in Table 3. Each of these tests is carried out to include an intercept and a time trend. Based on our empirical results, we find a uniform conclusion that the null hypothesis of non-stationarity can be strongly rejected at a 1% significance level. Therefore, we can conclude that all variables are stationary.

### 4.2. Results for Empirical Effects of the Credit Cycle on Happiness

In this part, our study explores the impact of the credit cycle on the reported happiness and particularly the varying effect of credit expansion and contraction on happiness. Thus, we estimate the regression specification (1)–(3) with economy fixed effect and year fixed effects for an unbalanced panel. The credit cycle is the main explanatory variable, while GDP per capita, healthy life expectancy, social support, freedom, generosity, corruption perception, positive affect, and negative affect are used as control variables. The results of the regression specification (1)–(3) are presented in Table 4 by utilizing the ordinary least squares linear regression (OLS).

In general, the credit cycle has a negative correlation with the happiness score, which confirms the Hypothesis 1 of our study. As shown in the second column of Table 2, the coefficient of the impact of the credit cycle on happiness is −0.0019, which is significant at the 1% level. This result suggests that a country’s credit growth will reduce the overall happiness score in a country. The reason can be drawn from the conclusion of Gudmundsdóttir et al. [58] that credit growth is accompanied by the rise of housing prices, the increase in luxury consumption and the prosperity of the gambling industry. This credit growth has severely hit the basis of the national economy and increased the probability of economic collapse in the entire country. In the different phases of the credit cycle, the impact of the credit cycle on happiness does not change the direction, however, the regression coefficient is less during credit recession than during credit expansion. As shown in column 3, the coefficient of the impact of the credit cycle on happiness during the expansion phase is −0.0012. And column 4 shows that the coefficient in credit recession is −0.0032. During the recession phase of the credit cycle, credit has a greater impact on the national happiness score than the expansion phase. Bank loans have a huge negative impact on individuals and reduce happiness by increasing residents’ financial stress and worries [61]. This result provides a shred of evidence that the national perception of happiness is more likely to feel the effect of credit contraction. For policymakers who are committed to improving national happiness, the negative impact of credit recession on national happiness is even more difficult to offset. Not only that, but the credit cycle has a stronger explanation of happiness during the recession phase (adjustment $R^{2}$ is 0.7270). The findings of this part bear further implications on the social sciences. The credit cycle has a negative correlation with national happiness. In particular, the credit contraction has a greater influence on national happiness and stronger explanatory power. This means that social science researchers should pay more attention to the period of credit recession or economic downturn when studying the impact of credit on happiness.

## 5. Moderating Factors between the Credit Cycle and Happiness

As the credit cycle has a negative impact on happiness, the next step in our study is to explore the transmission channels of the credit cycle on happiness. The empirical results in this part need to answer the question “Why the credit cycle has a negative impact on happiness”. In the study of Bhuiya et al. [91], the debt can indirectly influence the life satisfaction of micro-borrowers through a number of channels, such as the increased levels of worry. Since the credit cycle has a different effect on happiness during the period of expansion and recession, we should also pay attention to the influence of credit on happiness in different stages of the credit cycle in the following empirical evidence. To investigate whether the relationship between the credit cycle and happiness changes over time, we verify the moderating hypotheses 2a, 2b, and 2c of our study by estimating the regression specification (4). The main influencing factors of happiness, such as GDP per capita, are added to the regression specification (4) as moderating variables, thus obtaining 8 estimation results of panel regression models. In addition, the data needs to be processed before the panel regression model is estimated. The observed values of the explanatory variable (the credit cycle) and the moderating variable minus their arithmetic average. Friedrich [92] proved that this approach can get a standardized coefficient for the interaction term. Table 5 and Table 6 show the results of the panel regression after adding a variety of moderating variables during credit expansion and recession.

The moderating effects between the credit cycle and happiness are different during credit expansion and recession. As shown in Table 5, healthy life expectancy and generosity can moderate the relationship between the credit cycle and happiness only during the expansion of the credit cycle. The interaction terms of the credit cycle and healthy life expectancy, generosity are −0.0008, 0.0071. They are statistically significant at the 1% level. Given the interaction effect of healthy life expectancy and the credit cycle, the regression coefficient of the credit cycle become −0.0008∗Life+0.0537 By solving this equation, it can be concluded that when life expectancy is less than 67.1250, the overall effect of the credit cycle on happiness is positive. When life expectancy is greater than 67.1250, the overall effect of the credit cycle on happiness is negative. In other words, credit growth tends to lower the average happiness score when life expectancy is greater than 67.1250. However, in countries with higher generosity scores, credit growth will help to obtain higher average happiness scores. The result can be reasonably explained that government debt affects the social welfare system. When the government faced high debt and budget constraints, the price of municipal bonds fell, yields rose, and financing costs rose, forcing the government to reduce spending. These cuts are mainly concentrated on public welfare expenditures [93]. In countries with a high average life expectancy, the higher the debt they carry, the more likely they are to reduce government satisfaction. On the other side, credit growth indirectly increases the generosity of society by stimulating economic growth. In an environment of economic prosperity, people would tend to increase prosocial spending—spend money on others to get their own satisfaction [65].

GDP per capita can moderate the relationship between the credit cycle and happiness only during the recession of the credit cycle. As shown in Table 6, the interaction terms of the credit cycle and GDP per capita is 0.0019. It is statistically significant at the 5% level. This means that when a country’s GDP per capita is higher, greater credit helps to achieve a higher happiness score. Credit growth in a country/region may increase the profit rate, possibly resulting in higher investment, and then less unemployment [94]. In addition, credit growth means an increase in the level of financialization in this country/region. Bumann and Lensink [95] suggest that financial liberalization would improve the income distribution and resource allocation of this country. It led to lower the level of inequality in this country. Importantly, the study of Mikucka et al. [96] found that reduced income inequality would significantly improve subjective well-being in richer countries.

Whether the credit cycle is during expansion or recession phases, social support, freedom and positive affect can moderate the relationship between the credit cycle and happiness. During credit expansion, the interaction terms of the credit cycle and social support, freedom, and positive affect are 0.0519, 0.0185, and 0.0222, respectively. Moreover, during credit recession, the interaction terms of the credit cycle and social support, freedom, and positive affect are 0.0358, 0.0173, and 0.0223, respectively. They are all statistically significant at the 1% level. The results indicate that enhanced interaction effects between the credit cycle and social support, freedom, and positive affect can significantly improve happiness scores. The incidence of mental disorders can be reduced during the periods of credit growth, which is mainly due to the support from society and the family [58]. People with satisfactory social relationships are more likely to be happy, less sad, and more likely to improve their happiness [76]. In addition, the credit can improve quality of life when people have adequate social support [77]. For freedom, economic freedom is associated with smaller credit fluctuations and shorter credit recovery times [82]. Economic freedom improves the credit dilemma in a country/region and reduces pressure and worries from the debt. Finally, when positive affect spreads in financial markets, high social expectations would push the loans from banks to other investments, pursuing higher returns and increasing subjective well-being.

**Figure 1:**
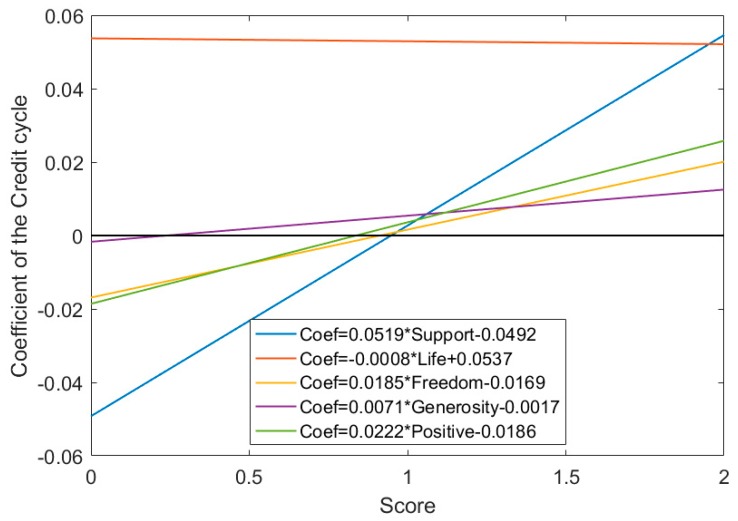
The total effect of the credit cycle on happiness during the expansion phase. α+λModerate denote the regression coefficient of the credit cycle term which represents the total effect of the credit cycle on happiness. ‘Life’ represents healthy life expectancy; ‘Support’ represents social support; ‘Positive’ represents positive affect. The sample period is from 2006 to 2018.

**Figure 2:**
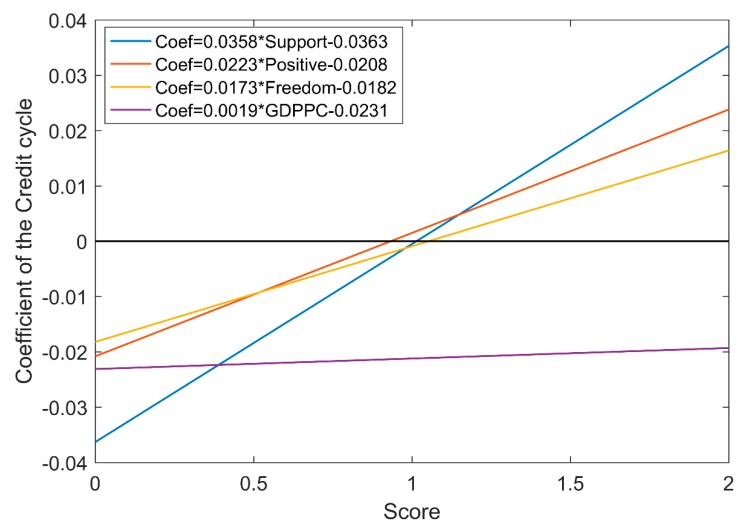
The total effect of the credit cycle on happiness during the recession phase. α+λModerate denote the regression coefficient of the credit cycle term which represents the total effect of the credit cycle on happiness. ‘GDPPC’ represents GDP per capita; ‘Support’ represents social support; ‘Positive’ represents positive affect. The sample period is from 2006 to 2018.

Next, we turn to the regression specification (5) which a simple conversion of the regression specification (4) to more directly express the total effect of the credit cycle on happiness. The regression specification (5) that divides the impact of the credit cycle on happiness into a part that can be explained by the direct effect and a part attributed to the changes in the indirect effects of these moderating variables on the relationship between the credit cycle and happiness. To investigate whether the credit cycle has a chance to increase happiness, we verify the moderating hypotheses 3 of our study by estimating the regression specification (5). We mainly discuss the regression coefficient of the credit cycle term which is denoted by α+λModerate. The regression coefficient of the credit cycle term represents the total effect of the credit cycle on happiness. Figure 1; Figure 2 show the results of the total effect of the credit cycle on happiness during expansion and recession phases.

The total impact of the credit cycle on the happiness index will become positive by the changes in the moderating effects of the moderating variable. During the expansion of the credit cycle, social support, freedom, generosity, and positive affect have a significant positive moderating effect on the relationship between the credit cycle and happiness. As shown in Figure 1, the impact of the credit cycle on happiness shifted from negative to positive if the score of social support reached 0.9496 (obtained by solving the equation Coef=0.0519∗Support−0.0492); or the score of freedom reached 0.9151 (obtained by solving the equation Coef=0.0185∗Freedom−0.0169); or the score of generosity reached 0.2412 (obtained by solving the equation Coef=0.0071∗Generosity−0.0017); or the score of the positive affect reaches 0.8372 (obtained by solving the equation Coef=0.0222∗Positive−0.0186). Life expectancy has a significant but weak negative moderating effect on the relationship between the credit cycle and happiness. In general, during the expansion of the credit cycle, we can improve subjective well-being if one of the following four conditions holds: (1) the adequate support from the family and society, (2) enough freedom, (3) social generosity, (4) with positive and optimistic outlook.

During the recession of the credit cycle, GDP per capita, social support, freedom, and positive affect have a significant positive moderating effect on the relationship between the credit cycle and happiness. As shown in Figure 2, the impact of the credit cycle on happiness shifted from negative to positive if the score of the natural logarithm of GDP per capita reached 12.7242 (obtained by solving the equation Coef=0.0019∗GDPPC−0.0231). However, the maximum natural logarithm of GDP per capita is only 11.4608 according to Table 2 Descriptive statistics. In addition, the impact of the credit cycle on happiness shifted from negative to positive if the score of social support reached 1.0154 (obtained by solving the equation Coef=0.0358∗Support−0.0363); or the score of freedom reached 1.0501 (obtained by solving the equation Coef=0.0173∗Freedom−0.0182); or the score of the positive affect reaches 0.9343 (obtained by solving the equation Coef=0.0223∗Positive−0.0208). But the scores of social support and freedom will not exceed 1. Finally, during the recession of the credit cycle, only with a high level of GDP per capita or positive and optimistic, the negative impact of credit growth on happiness can be offset. This is consistent with the above empirical results that the negative impact of credit recession on national happiness is difficult to offset even with an addition of a moderating effect.

Although there has been previous literature in the relationship between credit (or debt) and happiness, one contribution of our study is that the impact of credit on happiness can be changed from negative to positive under the influence of regulatory variables. This study does not doubt the predecessors’ conclusions: debt has a negative impact on happiness [49,50,51]. We investigate the relationship between credit and happiness from a macro perspective and give further policy implications to improve subjective well-being based on the negative correlation between the credit cycle and happiness. Social support, freedom, and positive affect as additional determinants of happiness throughout the credit cycle. We need to pay special attention to the moderating variables during the period of credit recession which can help to hedge the negative impact of credit growth on happiness.

## 6. Conclusions

Surveys from the international agencies show that during the 2008 international financial crisis, people’s happiness fell sharply and then fell into a “happiness trough”. At the same time, credit-to-GDP reached a peak in the third quarter of 2009. Previous literature put forward the “economics of happiness” theory, which triggered a heated discussion about happiness and economic. In this study, we test and demonstrate the relationship between the credit cycle and happiness using the fixed effects model. In addition, we explore the transmission channels of the credit cycle on happiness by adding the moderating effect. In particular, based on annual data for 42 economies in 2006–2018, we find the following empirical regularities. First, the credit cycle has a negative correlation with the happiness score. This means a country’s credit growth will reduce the overall happiness score in a country. The reason can be drawn from the conclusion of Gudmundsdóttir et al. [58] that credit growth is accompanied by the rise of housing prices, the increase in luxury consumption and the prosperity of the gambling industry, which has severely hit the basis of the national economy and increased the probability of economic collapse in the entire country. Second, the transmission channels between the credit cycle and happiness are different during credit expansion and recession. Healthy life expectancy and generosity can moderate the relationship between the credit cycle and happiness only during the expansion of the credit cycle. GDP per capita can moderate this relationship only during the recession of the credit cycle. Whether the credit cycle is during expansion or recession phases, social support, freedom, and positive affect can moderate this relationship. Third, the total impact of the credit cycle on happiness will become positive by the changes in the moderating effects of the moderating variable. Based on our empirical results, we confirm the previous finding on the negative impact of credit (or debt) on happiness [49,50,51]. However, one contribution of our study is that the impact of credit on happiness can be changed from negative to positive under the influence of regulatory variables.

Our findings present important implications for public mental health. In the context of fluctuations in the credit cycle, the national perception of happiness is inevitably affected by the negative impact of credit, and a good strategy for avoiding negative shocks can significantly improve subjective well-being. We find that the recession of the credit cycle is a better explanation of the decline in happiness than expansion. Therefore, social science researchers should pay more attention to the period of credit recession or economic downturn when studying the relationship between credit (or debt) and happiness. On the other side, social support, freedom and positive affect as additional determinants of happiness throughout the credit cycle. We need to pay special attention to the moderating variables during the period of credit recession which can help to hedge the negative impact of credit growth on happiness.

Our work is not without limitations, some of which open new avenues for future research. First, this article does not distinguish between long-term or short-term effects on the relationship between the credit cycle and happiness. Second, we used a single measurement for the dependent and independent variables. Third, this study assumes that happiness is measured on a cardinal scale. The happiness score used in this study is the national-level average scores in a country/region. It fails to pose a national-level average happiness score with 7 or 5 categories or with verbal labels, such as “very happy/happy/so-so/somewhat unhappy/very unhappy”. Thus, as a first extension, further research could examine the short- and long-term effects of the credit cycle on happiness. As a second extension, future research could study whether the impact of the credit cycle on happiness is spatially heterogeneous. On this subject, the World Happiness Report 2019 pointed out that the evolution of happiness is very different in the ten global regions.

## Figures and Tables

**Table 1:** Variables and database.

Type	Variable	Measurement	Range	Data Source
Explained variable	Happiness	Imagine a ladder, with steps numbered from 0 at the bottom to 10 at the top. The top of the ladder represents the best possible life for you and the bottom of the ladder represents the worst possible life for you. On which step of the ladder would you say you personally feel you stand at this time.	[0, 10]	GWP
Explanatory variables	The credit cycle	Credit-to-GDP.	-	BIS
Control variables and moderating variables	GDP per capita	GDP per capita is in terms of Purchasing Power Parity (PPP) adjusted to constant 2011 international dollars. This study uses the natural logarithm of GDP per capita, as this form fits the data significantly better than GDP per capita.	-	WDI
	Healthy life expectancy	Healthy life expectancies at birth.	-	WHO
	Social support	National average of the binary responses (either 0 or 1) to the GWP question “If you were in trouble, do you have relatives or friends you can count on to help you whenever you need them, or not?”	[0, 1]	GWP
	Freedom	National average of responses to the GWP question “Are you satisfied or dissatisfied with your freedom to choose what you do with your life?”	[0, 1]	GWP
	Generosity	Residual of regressing national average of response to the GWP question “Have you donated money to a charity in the past month?” on GDP per capita.	[0, 1]	GWP
	Corruption perception	National average of the survey responses to two questions in the GWP: “Is corruption widespread throughout the government or not” and “Is corruption widespread within businesses or not?” The overall perception is just the average of the two 0 or 1 responses. In case the perception of government corruption is missing, we use the perception of business corruption as the overall perception.	[0, 1]	GWP
	Positive affect	Average of three positive affect measures in GWP: happiness, laughter and enjoyment in the Gallup World Poll waves 3–7. These measures are the responses to the following three questions, respectively: “Did you experience the following feelings during a lot of the day yesterday? How about Happiness?”, “Did you smile or laugh a lot yesterday?”, and “Did you experience the following feelings during a lot of the day yesterday? How about Enjoyment?”	[0, 1]	GWP
	Negative affect	Average of three negative affect measures in GWP. They are worry, sadness and anger, respectively the responses to “Did you experience the following feelings during a lot of the day yesterday? How about worry?”, “Did you experience the following feelings during a lot of the day yesterday? How about sadness?”, and “Did you experience the following feelings during a lot of the day yesterday? How about anger?”	[0, 1]	GWP

Note: The variable “happiness” in this article was measured by the national-level average subject well-being score. The measurements shown in this table all refer to the World Happiness Report 2019 [88], except for the credit cycle. GWP denotes Gallup World Poll; BIS denotes Bank for International Settlements; WDI denotes World Development Indicators; WHO denotes World Health Organization.

**Table 2:** Descriptive statistics.

	Mean	Maximum	Minimum	Std. Dev.	Skewness	Kurtosis
Happiness	6.4568	7.9709	3.6607	0.8792	−0.5705	2.4814
Creditcycle	144.4800	420.0500	17.4750	79.9738	0.6621	3.4583
GDPPC	10.2763	11.4608	8.2158	0.5991	−0.7875	3.6908
Support	0.8913	0.9873	0.5106	0.0714	−2.3034	9.8669
Healthylife	69.1217	76.5000	48.6400	4.8412	−1.5112	5.5564
Freedom	0.8053	0.9698	0.3692	0.1238	−1.1236	3.9078
Generosity	0.0487	0.5456	−0.3364	0.1940	0.1754	2.1439
Corruption	0.6491	0.9833	0.0352	0.2514	−0.5526	1.9889
Positive	0.7701	0.9344	0.4347	0.0818	−1.1384	4.5934
Negative	0.2404	0.4822	0.1109	0.0614	0.5895	3.0177

Note: This table summarizes descriptive statistics (sample mean, maximum, minimum, standard deviation, skewness, kurtosis) of happiness, the credit cycle (Creditcycle), GDP per capita (GDPPC), healthy life expectancy (Healthylife), social support (Support), freedom, generosity, corruption perception (Corruption), positive affect (Positive), and negative affect (Negative) in 42 sample economies. The sample period is from 2006 to 2018.

**Table 3:** The results of unit root test for all variables.

Variable	Fisher-ADF	Fisher-PP
Happiness	173.4271 ***	173.4271 ***
Creditcycle	97.1850 *	97.1850 *
GDPPC	178.2402 ***	178.2402 ***
Support	294.2496 ***	294.2496 ***
Healthylife	155.8640 ***	155.8640 ***
Freedom	293.9268 ***	293.9268 ***
Generosity	297.6674 ***	297.6674 ***
Corruption	152.7710 ***	152.7710 ***
Positive	237.2338 ***	237.2338 ***
Negative	194.7823 ***	194.7823 ***

Note: This table summarizes unit-root tests for happiness, the credit cycle (Creditcycle), GDP per capita (GDPPC), healthy life expectancy (Healthylife), social support (Support), freedom, generosity, corruption perception (Corruption), positive affect (Positive) and negative affect (Negative) in 42 sample economies. Fisher-ADF denotes ADF unit-root tests; Fisher-PP denotes Phillips–Perron unit-root tests. *** and * indicate significance at the 1 and 10 percent levels respectively. The sample period is from 2006 to 2018.

**Table 4:** The effect of the credit cycle on happiness.

Dependent Variable = Happiness	(1)	(2)	(3)
Credit cycle	−0.0019 ***		
	−0.0005		
Expansion		−0.0012 *	
		(0.0007)	
Recession			−0.0032 ***
			(0.0007)
Control var.	Included	Included	Included
Economy fixed eff.	Included	Included	Included
Year fixed eff.	Included	Included	Included
Num. of econ.	42	42	42
Num. of obs.	488	288	200
Adjusted R^2^	0.7073	0.6879	0.7270

Note: This is a regression with economy fixed effect and year fixed effects for an unbalanced panel explaining annual national average happiness index in full sample economy responses from World Happiness Reports from 2006 to 2018. To save space, coefficients on the control variable are not reported, but the results of the control variable are available upon request. ‘Expansion’ and ‘Recession’ denote expansion and recession phases of the credit cycle, respectively. Coefficients are reported with robust standard errors clustered by economy in parentheses. ‘var.’ denotes variable; ‘eff.’ denotes effect; ‘econ.’ denotes economy; ‘Num.’ denotes number; ‘obs.’ denotes observation; *** and * indicate significance at the 1 and 10 percent levels respectively. The sample period is from 2006 to 2018.

**Table 5:** The results of the moderating effect between the credit cycle on happiness during credit expansion.

Dep. var. = Happiness	Moderate var.							
	= GDPPC	= Support	= Life	= Freedom	= Generosity	= Corruption	= Positive	= Negative
Panel A. Expansion								
Creditcycle	−0.0097	−0.0492 ***	0.0537 ***	−0.0169 ***	−0.0017 **	−0.0005	−0.0186 ***	0.0008
	(0.0083)	(0.0070)	(0.0125)	(0.0043)	(0.0007)	(0.0014)	(0.0043)	(0.0021)
Moderate var.	−0.0748	−1.0851	0.1152 ***	−1.2648 *	−0.9708 ***	−1.0000 **	−0.1565	1.4096
	(0.1541)	(0.9166)	(0.0180)	(0.6872)	(0.3441)	(0.4270)	(0.7599)	(1.2297)
Interaction	0.0008	0.0519 ***	−0.0008 ***	0.0185 ***	0.0071 ***	−0.0014	0.0222 ***	−0.0088
	(0.0008)	(0.0076)	(0.0002)	(0.0051)	(0.0023)	(0.0024)	(0.0054)	(0.0089)
Other var.	Included	Included	Included	Included	Included	Included	Included	Included
Economy fixed eff.	Included	Included	Included	Included	Included	Included	Included	Included
Year fixed eff.	Included	Included	Included	Included	Included	Included	Included	Included
Num. of econ.	42	42	42	42	42	42	42	42
Num. of obs.	288	288	288	288	288	288	288	288
Adjusted R^2^	0.6878	0.7374	0.7098	0.7028	0.6981	0.6869	0.7069	0.6878

Note: This is a regression with economy fixed effect and year fixed effects for an unbalanced panel explaining annual national average happiness index responses from World Happiness Reports from 2006 to 2018. To save space, coefficients on the other control variable are not reported, but the results of the control variable are available upon request. Coefficients are reported with robust standard errors clustered by economy in parentheses. ‘Creditcycle’ represents the credit cycle; ‘GDPPC’ represents GDP per capita; ‘Healthylife’ represents healthy life expectancy; ‘Support’ represents social support; ‘Corruption’ represents corruption perception; ‘Positive’ represents positive affect; ‘Negative’ represents negative affect; ‘var.’ denotes variable; ‘eff.’ denotes effect; ‘econ.’ denotes economy; ‘Num.’ denotes number; ‘obs.’ denotes observation; ***, **, and * indicate significance at the 1, 5, and 10 percent levels respectively. The sample period is from 2006 to 2018.

**Table 6:** The results of the moderating effect between the credit cycle on happiness during credit recession.

Dep. var. = Happiness	Moderate var.							
	= GDPPC	= Support	= Life	= Freedom	= Generosity	= Corruption	= Positive	= Negative
Panel B. Recession								
Creditcycle	−0.0231 **	−0.0363 ***	0.0224	−0.0182 ***	−0.0034 ***	0.0015	−0.0208 ***	0.0022
	(0.0096)	(0.0093)	(0.0148)	(0.0058)	(0.0007)	(0.0016)	(0.0064)	(0.0026)
Moderate var.	0.2790	1.1849	0.0605 ***	−0.9782	0.3365	0.6946	−0.3907	4.7287 ***
	(0.1987)	(1.2635)	(0.0224)	(0.8002)	(0.4085)	(0.5111)	(1.1791)	(1.4927)
Interaction	0.0019 **	0.0358 ***	−0.0004	0.0173 ***	0.0038	−0.0085	0.0223 ***	−0.0228
	(0.0009)	(0.0100)	(0.0003)	(0.0066)	(0.0028)	(0.0056)	(0.0081)	(0.0207)
Other var.	Included	Included	Included	Included	Included	Included	Included	Included
Economy fixed eff.	Included	Included	Included	Included	Included	Included	Included	Included
Year fixed eff.	Included	Included	Included	Included	Included	Included	Included	Included
Num. of econ.	42	42	42	42	42	42	42	42
Num. of obs.	200	200	200	200	200	200	200	200
Adjusted R^2^	0.7323	0.7452	0.7301	0.7363	0.7283	0.7416	0.7375	0.7327

Note: This is a regression with economy fixed effect and year fixed effects for an unbalanced panel explaining annual national average happiness index responses from World Happiness Reports from 2006 to 2018. To save space, coefficients on the other control variable are not reported, but the results of the control variable are available upon request. Coefficients are reported with robust standard errors clustered by economy in parentheses. ‘Creditcycle’ represents the credit cycle; ‘GDPPC’ represents GDP per capita; ‘Healthylife’ represents healthy life expectancy; ‘Support’ represents social support; ‘Corruption’ represents corruption perception; ‘Positive’ represents positive affect; ‘Negative’ represents negative affect; ‘var.’ denotes variable; ‘eff.’ denotes effect; ‘econ.’ denotes economy; ‘Num.’ denotes number; ‘obs.’ denotes observation; *** and ** indicate significance at the 1 and 5 percent levels respectively. The sample period is from 2006 to 2018.
